# Towards a cyber-physical framework for crime prevention: An architect’s Perspective

**DOI:** 10.1192/j.eurpsy.2026.10477

**Published:** 2026-07-31

**Authors:** E. Abdelmoula, N. Bouayed Abdelmoula, B. Abdelmoula

**Affiliations:** 1 LR21ES19 UMRAN, National School of Architecture and Urbanism of Tunis; 2 Doctoral school of architectural sciences and engineering (ED-SIA), Carthage University, Carthage; 3 LR23ES07 Genomics of Signalopathies at the service of Precision Medicine, Medical University of Sfax, Sfax, Tunisia

## Abstract

**Introduction:**

Architectural design plays an essential role in creating safe and inclusive urban environments. While the CPTED (Crime Prevention Through Environmental Designprinciples) provide fundamental strategies for crime prevention through environmental design, the rise of digital modeling platforms such as Building Information Modeling (BIM) and City Information Modeling (CIM) offers new opportunities to simulate and predict crime dynamics before implementation. The integration of behavioral data, in particular from psychiatric and social sciences, can further improve the understanding of territorial social behaviors, essential for an effective deterrence of crime.

**Objectives:**

This study aims to propose a formalized interdisciplinary framework that integrates BIM, CIM, CPTED principles and behavioral information to allow architects to predict, evaluate and design crime-resilient environments. The objective is to establish a systematic approach to integrate physical, social and psychological factors into the architectural design process in order to optimize the safety and well-being of the community.

**Methods:**

The development of the framework involves a literature review of CPTED, BIM, CIM and behavioral sciences models, followed by a conceptual mapping of data flows and interactions. Hypothetical modeling scenarios show how architectural design parameters can be linked to agent-based social simulations and predictive analytics. The interdisciplinary collaboration between architects, urban planners and psychiatrists sheds light on the design of integrated workflows.

**Results:**

The preliminary formalization reveals that the coupling of BIM /CIM data with CPTED strategies and psychosocial behavioral modeling allows an improved spatial analysis of risk areas, a better prediction of crime hotspots and optimized environmental interventions. The model supports iterative design changes based on simulated social dynamics and safety outcomes, suggesting an important potential of improvement in design efficiency compared to traditional CPTED-only approaches.

**Conclusions:**

The integration of BIM, CIM and CPTED with psychiatric and social behavioral data offers architects a powerful data-driven tool to design safer and more adaptive urban environments. This formalized framework encourages multidisciplinary collaboration and provides a basis for future empirical validation and practical implementation in various urban contexts.

**Disclosure of Interest:**

None Declared

